# A longitudinal perspective on the interplay of job demands and destructive leadership on employees’ work ability in Germany

**DOI:** 10.1007/s00420-023-01962-z

**Published:** 2023-03-25

**Authors:** Carolin Kunz, Catrin Millhoff

**Affiliations:** TU Dortmund University / Technische Universität Dortmund, Dortmund, Germany

**Keywords:** Job demands–control (JDC) model, Work ability, Destructive leadership

## Abstract

**Purpose:**

Work ability as a predictor of early retirement or lengthy/frequent sick leaves becomes more and more relevant due to the demographic change. Therefore, factors, which affect employees’ work ability, need to be further examined with a theoretical base. According to Karasek’s job demands–control (JDC) model, high job demands and low control are related to poor employee health. The subsequent job demands–control support (JDCS) model proposed that a lack of support, also from leaders, has a negative impact on health indicators. This article looked at whether destructive leadership reinforces the negative influence of high job demands on employees’ work ability.

**Methods:**

We used the BAuA Working Time Survey waves 2015, 2017, and 2019 (BAuA-WTS), which are representative of employees in Germany. Our data set covers 2448 respondents, who took part in all three waves. Central to the analyses were the variables job demands, control, destructive leadership, and work ability.

**Results:**

Results from pooled ordinary least squares (OLS) and fixed effect (FE) models revealed that job demands influenced work ability negatively, whereas neither control by the employee nor the interaction of job demands and control was significant regarding effecting work ability in the FE models. Destructive leadership exerted a negative effect on work ability and moderated job demands significantly in the FE models meaning a further decrease in work ability.

**Conclusion:**

Leadership behavior should be further examined as an important factor for work ability.

## Introduction

Due to the demographic change, maintaining the work ability of (older) employees becomes more and more relevant for research but also for organizations because a low work ability may lead to e.g., early retirement (Tisch 2015). This means a loss of organizational human capital for organizations and lower (financial) resources for individuals. The concept of “work ability” is a concept covering a balance of employees’ health, personal resources, working conditions and ability to cope with job demands (Ilmarinen 2001). Given the relevance of work ability for predicting important organizational criteria, such as retirement age, sick leave, or future physical/mental health limitations, this concept has become the focus of increased research (for a review, see Cadiz et al. 2019). Several studies have shown that work ability depends on organizational factors, such as job demands (Brady et al. 2020; Burr et al. 2022; McGonagle et al. 2014) and work resources, including supervisor support (Brady et al. 2020; Burr et al. 2022; Elo et al. 2008).

The relationship between job demands and health is widely examined by means of the job demands–control (JDC) model (Karasek 1979) and its expanded version, the job demands–control–support (JDCS) model (Johnson et al. 1989), in which the social component “support” was introduced. Having high job demands in combination with low levels of control on the employee’s part can lead to health impairments (van der Doef and Maes 1998). In the JDCS model, high demands, low control, and social isolation characterize the most detrimental working conditions when it comes to health outcomes (e.g., cardiovascular diseases) (van der Doef and Maes 1998). Although the models also describe a buffering hypothesis, which suggests that high job demands can be buffered by the presence of greater control and/or support, previous studies have been unable to find clear support for either of these hypotheses (Luchman and González-Morales 2013; van der Doef and Maes 1998).

The role of leadership has already been integrated into other job strain models (Schaufeli 2015), but there is still a gap in the research concerning the JDC model and its assumed correlations. Numerous studies have demonstrated that supportive leadership (e.g., transformational leadership) enhances motivation and performance (Wang et al. 2011) and promotes health (Zwingmann et al. 2014). In contrast, both the passive (laissez-faire) and active (abusive leadership) forms of destructive leadership can have a notably negative impact on employees’ health (Schyns and Schilling 2013). These factors have been previously connected with work ability (Brady et al. 2020) but not examined with respect to the JDC(S) model and especially age.

Accordingly, we examined the influences of work demands for predicting work ability within the framework of the JDC(S) model by asking the following question: “Are a rise in job demands, a decrease in job control, and a shift toward destructive leadership associated with a reduced work ability? And does a combination of adverse working conditions intensify the negative effect on work ability?” In detail, we will explore the question of whether destructive leadership behavior intensifies the negative relationship between job demands and work ability, since such behavior can be considered a stressor. To investigate this connection, we used three waves of the BAuA–Working Time Survey (BAuA-WTS) of the years 2015, 2017, 2019 (BAuA 2019a, b, 2021), which provided a representative sample of employees in Germany (Häring et al. 2016, 2018, 2020).

The article begins with a short overview of the state of research on work ability. We then present the JDC model and add the theoretical component “leadership” explaining why leadership is an important factor in working conditions and may contribute to the prediction of work ability. Next, our resulting hypotheses are introduced before we offer insights concerning the data obtained from our 2,448 survey respondents, who answered in each wave. We analyzed the data by comparing ordinary least squares (OLS) and fixed effect (FE) models. FE models are a frequently used method, where intra-individual differences are examined. The descriptive and longitudinal results follow and are then discussed along with theoretical and practical implications for future research.

## Theoretical framework

### Work ability

In the 1980s, the concept of work ability was developed by the Finnish Institute of Occupational Health. This concept represents employees’ ability to fulfill their job demands according to their human resources (Ilmarinen 1999). Fundamental to work ability is a person’s health, including physical, mental, and social capacities. Other necessary human resources are education and competence, values, attitudes, and motivation. A person’s work ability is high if these resources are in balance with working conditions (Ilmarinen 2001). Work ability may serve as an indicator of the likelihood of early retirement or extended periods of sick leave. According to data from the German lidA Cohort Study on Work, Age and Health, which included 3,796 participants 46 years of age or older, self-perceived work ability had a direct effect on participants’ decision to end their employment (Tisch 2015).

Work ability is a dynamic concept: On the one hand, human resources change, particularly as an employee ages, and physical and mental capacities decline (van den Berg et al. 2008). On the other hand, work and working conditions (e.g., new technology) are in flux, which can challenge aging employees (Ilmarinen 2001). Therefore, work ability depends strongly on the work environment: According to a systematic review of working conditions and the Work Ability Index, high mental and physical demands and low control were associated with poor work ability (van den Berg et al. 2008). The employee’s perception of management’s leadership quality is another component of working conditions that might affect work ability: Cross-sectional and longitudinal studies have shown a negative association between poor management and work ability (Tuomi et al. 2001, 1997). Another German study focused on the relationship among working conditions, work ability and depressive symptoms: In this study, decreasing demands and increasing leadership quality between the two waves were associated with higher work ability, while the effects regarding job control and social support were positive but rather small (Weber et al. 2021).

### Job demands–control model

Karasek’s (1979) JDC model is a widely applied and tested theoretical foundation for explaining the occurrence of strain due to working conditions (e.g., Rijk et al. 1998; van Yperen and Hagedoorn 2003). The JDC model characterizes work as having two components: job demands and control on the part of the employee. Job demands are understood to include such factors as quantitative workload and conflicting demands, while control comprises decision authority and autonomy over tasks. According to the JDC model, physiological strain results from a combination of high work demands and limited decision latitude and describes a highly stressful job (the job strain hypothesis). If high job demands can be compensated with more decision latitude, employees are able to organize the way their work is executed and can channel their energy into useful activities that allow them to release strain (the buffering or learning hypothesis). Karasek (1979) originally developed the model to predict physiological strain, but it has since been broadened to address effects on mental factors, such as burnout (Rijk et al. 1998), depression (Blackmore et al. 2007), or even mortality (Gonzalez-Mulé and Cockburn 2021), enabling an employee’s overall health status to be explained by the model.

Since health is considered a key determinant of work ability (Ilmarinen 2001), it can be assumed that work ability can also be explained using the JDC model. High demands reduce work ability, while control over tasks has a positive impact on work ability. In a Swedish study involving 7,810 respondents, male employees with low skill discretion—a component of control—had a lower work ability over the 7-year follow-up study (Leijon et al. 2017).

As far as the interplay between the different components of the JDC model is concerned, a meta-analytic review of 106 studies concluded that these interrelationships need to be further examined both theoretically and empirically. The originally proposed interaction between job demands and control often showed no significant effects on different outcomes (Luchman and González-Morales 2013). Overall, the state of research is equivocal (de Lange et al. 2003; Meier et al. 2008): Taris (2006) found that only 10% of the 63 studies of the JDC model reviewed by van der Doef and Maes (1998) supported the moderation of demand and control. Consequently, there is still a great need to test this model, which leads to our first three hypotheses:

*Hypothesis 1 (H1): Job demands have a negative effect on work ability.*

*Hypothesis 2 (H2): Job control has a positive effect on work ability.*

*Hypothesis 3 (H3): Job control moderates the negative relationship between job demands and work ability, such that the influence will be weaker when job control increase.*

### Demanding leadership behavior—the role of destructive leadership

The JDCS model proposed by Johnson et al. (1989) already included support but did not distinguish the sources of support (co-workers or leaders), thus preventing a more detailed look at the possible effects of leaders per se. Nevertheless, leaders play a central role when one is examining the effects of working conditions on employees' ability to work, because leadership behavior influences their perception of work (Fernet et al. 2015; Sparrowe and Liden 1997) and can actively shape working characteristics (Schaufeli 2015).

In practice, it becomes apparent that not only a lack of supportive leadership behavior (Aasland et al. 2010), but even hostile leadership behavior is often prevalent (Tepper et al. 2017). Thus, over the last decade, destructive leadership behavior has increasingly become the focus of leadership research owing to its negative impact on organizational outcomes (Mackey et al. 2021; Schyns and Schilling 2013). Destructive leadership has been found to have detrimental effects on such outcomes as job satisfaction, well-being and stress among employees (Schyns and Schilling 2013). In the long run, Schmidt et al. (2018) found that when such supportive leadership behaviors were lacking, employees' self-assessed health over a 10-year period was significantly poorer compared with that of employees who felt supported. In our study, destructive leadership is understood to include both actively negative leadership behavior such as abusive supervision (Tepper 2000), in keeping (partially) with the definitions of e.g., Shaw et al. (2011) and Skogstad et al. (2007).

In contrast to the transformational leader, who demonstrates to employees the importance of their work (Yukl 2010), thus improving their performance by increasing their self-worth and intrinsic motivation (Bass 1985), the destructive leader exhibits neglectful behavior by being absent or even displays hostile behavior through verbal abuse or unwarranted punishment, thus impairing employees’ performance and well-being (Tepper et al. 2017). According to Zwingmann et al. (2014), the health-promoting, stress-reducing effects of transformational leadership in particular could be attributed to this concept’s core characteristics, which include support, empowerment and a high-quality relationship between leaders and their employees. In addition, transformational leadership has a resource-saving function through its influence on employees’ self-concept (Shamir et al. 1993), which helps them cope with work demands. Related to the concept of destructive leadership and based on the JDCS model, such a stress-reducing effect is absent when no support is given and the relationship between leader and employees is of low quality (Buch et al. 2015). Considering destructive leadership, one can assume that destructive leaders strain the resources of their followers, considerably impairing employees' ability to work and exacerbating the effect of high job demands on work ability. This assumption is supported by a Dutch study involving 19,507 construction workers in which low levels of supervisor and co-worker support had negative effects on work ability (Alavinia et al. 2007). Similar results were reported in a study involving Australian health care workers (McGonagle et al. 2014).

*Hypothesis 4 (H4): Destructive leadership has a negative effect on work ability.*

Within the framework of the JDCS model (Johnson et al. 1989), leadership can be seen as the supportive component and is described as a factor that can mitigate tension due to less favorable working conditions, such as high job demands and low levels of control (Westerlund et al. 2010). Accordingly, leadership can be seen as a moderating influence in the relation between job demands and employees’ well-being. However, destructive leaders can negatively affect an employee’s self-concept (Vogel and Bolino 2020), potentially resulting in a loss of resources and greater difficulty in dealing with high work demands. In their recent systematic review of the leadership research, Tummers and Bakker (2021) point out that, only a few studies have examined the moderation of demands and leadership and that the results regarding such moderation were not consistent. Consequently, there is still a large research gap when it comes to the impact of leadership and its interplay with job demands with respect to work ability. To help address this gap, we investigated the following hypothesis:

*Hypothesis 5 (H5): Destructive leadership moderates the negative relationship between job demands and work ability, such that the influence will be stronger for employees with a destructive leader.*

## Materials and methods

### Data

For our analysis, we relied on the waves 2015, 2017, and 2019 of the scientific use file of the BAuA Working Time Survey (BAuA-WTS) (BAuA 2019a, b, 2021), a representative panel survey of dependent or self-employed employees conducted by the Federal Institute for Occupational Safety and Health (BAuA) in Germany. Respondents aged 15 years and older, who were employed for at least 10 h per week, were accessed via Computer-Assisted Telephone Interview (CATI). The mobile and landline numbers were generated randomly. About 20,000 interviews were conducted in 2015 and more than 10,000 interviews in 2017 and 2019. About 7,000 respondents participated in at least one wave (Brauner et al. 2019a, b; Häring et al. 2016, 2018, 2020; Pattloch et al. 2021). The survey included questions about working conditions but also about work ability and health.

To attain a more homogeneous sample, we included participants who had contractually agreed working hours of 10 h and more per week and whose monthly income exceeded €450. Respondents who were self-employed, family workers or working as freelancers were excluded. We obtained a balanced panel by excluding participants, who had missing values in at least one of the relevant variables. Consequently, each person took part in all three waves of data collection. Across all three waves, we used information on the work ability of 2448 respondents (7344 observations), the majority of whom were male (53.54%). Overall, the mean age was 48.91 years (SD = 9.03). On average, respondents reported 35.17 contractually agreed working hours and a monthly gross income of 3,595.69 €.

### Assessments and measures

#### Work ability

The original Work Ability Index (WAI) consists of multiple variables that were not included in the scientific use file of the BAuA-WTS. The use of a single-item question is recommended by e.g., Ahlstrom et al. (2010) because they showed a very strong correlation between the WAI and the single-item measure (*r* = 0.87). Besides, other studies, like e.g., Thorsen et al. (2013) used a single-item work ability measure to predict sickness absence. Finally, since the one-dimensionality of the variables within the Work Ability Index (WAI) is often criticized anyway (Martus et al. 2010), work ability was measured here by the item “Assume that your work ability at its best has a value of 10 points. How many points would you give your current work ability? (0 means that you currently cannot work at all)” and has a range from 0 to 10. The use of a single item such as this is rather common (van den Berg et al. 2008). The mean value of our metric dependent variable was 7.85 (SD = 1.71), indicating a rather high work ability score.

#### JDC model

The “demands” component of the JDC model was measured by six items: time pressure and pressure to perform, working fast, hiding feelings, confrontation with problems of other people, simultaneous handling of work sequences and interruptions by colleagues. These variables are similar to those in the original operationalization of the JDC model (Karasek 1979) and in studies in which the JDC model was applied to secondary data (e.g., de Jonge et al. 2000). The “control” component was covered by five variables: work execution specified, ability to plan his/her own work, influence on the amount of work, decision on point of time and frequency of breaks and influence on type of tasks. Scores for both the demands and the control variables ranged from 1 (“never”) to 4 (“frequently”). The questionnaire did not cover a pure version of the JDCS model but parts of the Copenhagen Psychosocial Questionnaire, the Effort-Reward Imbalance model and the German Short Work Analysis Questionnaire (Häring et al. 2016, 2018, 2020). We used a selection of items, which were in line with the JDCS model.

Before creating sum scores, we recoded each variable to obtain high values for high demands and high levels of control. The internal consistency across all waves was somewhat acceptable (Cronbach’s alpha_demands_ = 0.64; Cronbach’s alpha_control_ = 0.67) due to the dependence of Cronbach’s alpha on the number of variables considered (Bortz and Döring 2006). Yet, an index does not automatically need to be excluded as long as it has a strong theoretical foundation. To give a first overview in the descriptive statistics, we dichotomized the variables demands and control using the median as a cut-off value. Respondents above the median were categorized as high demands or high control. For the regression models with interaction effects of demands with control and demands with destructive leadership, we calculated z-scores for the variables demands and control.

#### Destructive leadership behavior

For this assessment, we used subjective measures, since employees’ perception of the quality of leadership is important (Shaw et al. 2011). Shaw et al. (2011) developed an instrument for measuring the dark side of leadership, which was based on a qualitative study on negative leadership behaviors conducted by Erickson et al. (2007). Since the data collected from the Federal Institute for Occupational Safety and Health (BAuA) did not focus on leadership, only one item addressing destructive leadership was available: leader exhibits inconsiderate behavior. This item matches the dimension “an inconsiderate tyrant,” (Shaw et al. 2011). Steinmann et al. (2020) used the BAuA-WTS and took inconsiderate behavior as a proxy variable for destructive leadership. Therefore, we also treated our measurement of leadership as an indicator for destructive leadership. We dichotomized the variable by categorizing 3 (“sometimes”) and 4 (“frequently”) as destructive leadership and 1 (“never”) and 2 (“rarely) as non-destructive leadership. Overall, 11.70% of the respondents reported destructive leadership. Across all three waves, 471 employees changed their perception from having a non-destructive leader to a destructive leader.

#### Control variables

As part of the JDCS model, we added co-worker’s support. Co-worker’s support was measured based on three items (feeling part of the workplace community, good teamwork and support by colleagues) and is also a sum score of the three individual items (Cronbach’s alpha_co-worker support_ = 0.64). Since work ability decreases with age (Ilmarinen 2001), we included the mean centered age of the respondents. We also added sex and the natural logarithm of the respondents’ monthly gross income. Income is a component of social status, which might in turn be related to the respondents’ health status (Lampert et al. 2019) and therefore presumably their work ability. At last, we added dichotomous variables for the wave to the regression models.

### Statistical analysis

At first, we used pooled OLS models to predict work ability by adding the independent variables stepwise. Regression models allow test hypotheses by analyzing the relations between a dependent variable and one or more independent variables. We included interaction effects, consisting of the product of the independent variable and the moderator. (Kohler and Kreuter 2016). The OLS models with robust standard errors were compared with FE models to analyze intra-individual variation. FE model is an advanced panel data method where the difference of a variable for each wave (t) per person (i) with the mean value of a variable for each wave per person is taken into consideration (Wooldridge 2013):

$$\left({y}_{it}-{\overline{y} }_{i}\right)={b}_{1}\times \left({x}_{it}-{\overline{x} }_{i}\right)+{b}_{2}\times \left({z}_{i}-{z}_{i}\right)+{w}_{it}$$

Since time-demeaned data is used, fixed effects models control for time-invariant variables (here shown as ${z}_{i}$) and therefore these models are not biased by unobserved factors, which are time constant. By means of the longitudinal models, selection factors and reverse causality can be detected (Wooldridge 2013). The data were prepared and the models were performed with use of the statistical program Stata 16.1.

## Results

Respondents with high demands reported that their work ability was lower (*M*_high demands_ = 7.57) than it was for those with low demands (*M*_low demands_ = 8.03), and this difference was significant (*p* ≤ 0.001). The significant difference also applied to the issues of control and leadership. Employees with low control (*M*_low control_ = 7.71 vs. *M*_high control_ = 8.06; *p* ≤ 0.001) and destructive leadership (*M*_destructive_ = 7.16 vs. *M*_not destructive_ = 7.94; *p* ≤ 0.001) perceived that their work ability was worse. Highly significant correlations among the variables supported this tendency (see Table 2 in the appendix). Respondents’ work ability was the lowest when job demands were high and destructive leadership was in place (*M*_high demands & destructive leader_ = 6.97) when compared with rather high work ability among employees with low job demands and no destructive leader (*M*_low demands & no destructive leader_ = 8.07). Although the descriptive results provided initial insights into the relationships among the variables and the possible moderated effect of leadership, it was important to test the hypotheses using multivariable analyses.

At first, we tested the general assumptions of the JDC model with regard to predicting work ability (see Table 1). In the pooled OLS model (model 1, adjusted *R*^*2*^ = 0.0659; *p* ≤ 0.001) and also in the FE model (model 4, within *R*^*2*^ = 0.0207; *p* ≤ 0.001), job demands were negatively related to work ability. An increase in job demands goes along with a decrease of respondents’ work ability, which is in line, with our first hypothesis (H1). Hypothesis 2 was only supported in the pooled OLS model. Respondents with higher levels of control reported higher work ability (*b* = 0.099; *p* ≤ 0.001). Yet, the effect was not significant in the fixed effects model, which indicates inter-individual and not intra-individual differences. Therefore, no causal effect was found and we had to reject the second hypothesis. Additionally, the interaction of demands and control—the central assumption of the JDC model—was neither significant in the OLS nor in the FEM model and had to be rejected (H3). In all models, the coefficients remained rather stable when the control variables were added (results not shown). Only co-worker support^1^ had a significant and positive effect on work ability in the FE models. Consequently, an increase in co-worker support leads to a higher work ability.

**Table 1:** Regression models for work ability

	Pooled OLS						FE					
	1		2		3		4		5		6	
	Coef	SE	Coef	SE	Coef	SE	Coef	SE	Coef	SE	Coef	SE
Demands (z)	− 0.163***	0.025	− 0.163***	0.025	− 0.150***	0.026	− 0.148***	0.035	− 0.149***	0.035	− 0.134***	0.035
Control (z)	0.099***	0.026	0.099***	0.026	0.099***	0.026	0.039	0.037	0.039	0.037	0.038	0.037
Demands × control	–	–	0.001	0.021	–	–	–	–	− 0.034	0.026	–	–
Destructive leadership (ref.: no)	− 0.453***	0.078	− 0.453***	0.078	− 0.372***	0.084	− 0.420***	0.076	− 0.426***	0.077	− 0.339***	0.087
Demands*destructive leadership	–	–	–	–	− 0.159^+^	0.085	–	–	–	–	− 0.164*	0.082
Co-worker support	0.154***	0.018	0.154***	0.018	0.154***	0.018	0.082***	0.021	0.083***	0.021	0.082***	0.021
Age (centered)	− 0.018***	0.003	− 0.018***	0.003	− 0.018***	0.003	0.016	0.037	0.017	0.037	0.016	0.037
Sex (ref.: male)	0.191***	0.055	0.191***	0.055	0.191**	0.055	–	–	–	–	–	–
Income (log.)	0.218***	0.053	0.218***	0.053	0.217***	0.053	0.177	0.116	0.174	0.116	0.178	0.116
Wave 2017 (ref.: 2015)	0.028	0.041	0.028	0.041	0.027	0.041	− 0.030	0.082	− 0.032	0.082	− 0.030	0.082
Wave 2019 (ref.: 2015)	− 0.114**	0.043	− 0.114**	0.043	− 0.116**	0.043	− 0.239	0.158	− 0.242	0.158	− 0.241	0.158
*R*^2^	0.0659***		0.0659***		0.0665***		–		–		–	
Within *R*^2^	–		–		–		0.0207 ***		0.0210***		0.0215***	
*n* (persons)	2448		2448		2448		2448		2448		2448	
*N* (observations)	7344		7344		7344		7344		7344		7344	

***p ≤ 0.001; **p ≤ 0.01; *p ≤ 0.05; ^+^*p* ≤ 0.10; coef. = unstandardized coefficients; SE = standard error; pooled OLS: clustered standard errors; BAuA Working Time Survey waves 2015, 2017 and 2019; balanced panel

As a result of these findings, we neglected the interaction of job demands and control in favor of focusing on leadership. The coefficient of destructive leadership is significant and negative in every model. According to the FE models, employees’ work ability decreases when respondents’ perception of leadership changes from a not destructive leader to a destructive leader, who behaves inconsiderately (e.g., model 4: *b* = − 0.420; *p* ≤ 0.001). Consequently, H4 was supported.

**Fig. 1:**
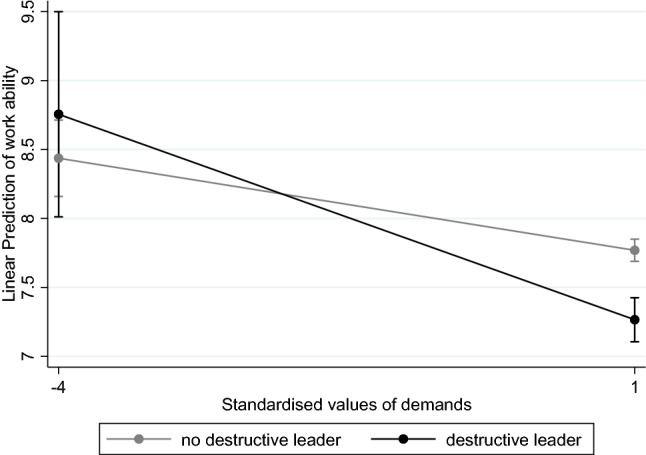
Predictive margins for work ability by destructive leadership (model 6, FEM)

As was assumed in our fifth hypothesis (H5), destructive leadership moderates the relationship of job demands and work ability. We found no significant interaction effect of job demands and destructive leadership cross-sectionally (model 3, *p* = 0.062) but in the FEM model (model 6: *b* = − 0.164;* p* ≤ 0.05). Thus, employees’ work ability worsened significantly when demands increased in combination with a shift to destructive leadership, which supports our fifth hypothesis (H5) (see Fig. 1).^2^

## Discussion

In this article, we applied the JDC model to work ability with a focus on destructive leadership. Our results supported the adverse effect of high job demands (H1), as was shown in the meta-analysis conducted by Luchman and González-Morales (2013). Their meta-analysis also revealed that the moderation of job demands and control was not significant in the majority of the studies, which was also the case in our study. Therefore, we had to reject our third hypothesis (H3). Contrary, we could not find a significant longitudinal effect of control on work ability. One reason for the deviation of our results might be that the meta-analysis by Luchman and González-Morales (2013) does not transparently distinguish between cross-sectional and longitudinal studies.

Moreover, we demonstrated that destructive leadership negatively influences work ability, as assumed in hypothesis 4 (H4). Employees shifting in a destructive leadership relationship perceived their work ability to be significantly lower. This finding is consistent with negative consequences of destructive leadership on e.g., well-being and stress of employees (Schyns and Schilling 2013). The combination of high job demands and destructive leadership has an even worse effect according to our fifth hypothesis (H5). Consequently, we conclude that a shift into a destructive leadership relationship in combination with an increase in job demands leads to an even lower work ability.

Our results have two important theoretical implications for future research. By integrating the research streams of industrial and organizational psychology and sociology, our findings support the assumption made by Weber et al. (2021) that leadership behavior is a crucial factor in maintaining employees' work ability. However, by focusing on destructive leadership behaviors, we were able to show its negative consequences for work ability, which is in line with previous research on the negative effects of destructive leadership on health (e.g., Schyns and Schilling 2013). These findings highlight the need to investigate further the relationship between leadership behaviors and work ability in light of demographic changes in the labor market. Yet, future research should include more items measuring destructive leadership like e.g., Shaw et al. (2011) in large surveys. Besides, physical job demands should also be considered in future longitudinal research because work ability is also influenced by this component as Burr et al. (2022) showed in a 5-year prospective study.

Previous research has shown that work ability plays a central role in employment decisions such as early retirement (Tisch 2015) and is therefore of key relevance to maintaining organizational human capital. The results of the present study underline that employees' work ability depends on organizational factors, working conditions and leadership behavior. These factors can be actively influenced by organizations and human resource management. Organizations should regularly conduct employee surveys to ensure that employees do not permanently face high job demands and destructive leadership, in this way, to prevent health impairments.

Using a large sample that can be considered representative at the national level (Häring et al. 2016), we were able to generalize our results to apply them to the most diverse occupations and to guide organizational decisions. Another strength of our study is the longitudinal approach. By means of FE models, we found evidence for causality that high job demands, destructive leadership and the interplay influence employees’ work ability negatively. However, our study also has limitations. The constructs job demands, control and co-worker support could have shown a higher internal consistency, which might be due to the scaling. However, this article focused on the application of the JDC model rather than on testing and refining the theory itself. Another limitation is the use of a single-item question for work ability and destructive leadership respectively. Although item sets—considering the multiple dimensions of both constructs—exist, we could not make use of them, because they were not included in the BAuA-WTS. However, the advantages of a representative survey predominated.

Overall, the analysis of job demands and destructive leadership is promising to predict employees’ work ability. Companies should therefore put maintaining the work ability of their older employees on their agenda to avoid early retirement or long periods of sick leave. As shown in this article, leadership and e.g., leadership development programs—including strategies to reflect their leadership style, dealing with stress, promoting self-regulation and positive leadership behavior like transformational leadership—could be a starting point.

## Data Availability

Bundesanstalt für Arbeitsschutz und Arbeitsmedizin (BAuA): https://doi.org/10.21934/BAUA.AZB15.SUF.1. https://doi.org/10.21934/BAUA.AZB17.SUF.1. https://doi.org/10.48697/BAUA.AZB19.SUF.1.

## Appendix

See Table 2.

**Table 2:** Inter-correlations

	Work ability	Demands	Control	Destructive leadership	Co-worker support	Age	Sex	Income (ln)
Work ability	1.000							
Demands (z)	− 0.119***	1.000						
Control (z)	0.108***	− 0.111***	1.000					
Destructive leadership (no vs. yes)	− 0.147***	0.206***	− 0.138***	1.000				
Co-worker support	0.171***	− 0.106***	0.107***	− 0.261***	1.000			
Age (mean centered)	− 0.100***	0.016	− 0.006	0.023*	− 0.033**	1.000		
Sex (male vs. female)	− 0.003	0.144***	− 0.146***	0.070***	0.034**	0.097***	1.000	
Income (ln.)	0.056***	0.092***	0.333***	− 0.062***	0.034**	0.053***	− 0.397***	1.000

****p* ≤ 0.001; ***p* ≤ 0.01; **p* ≤ 0.05

## Footnotes

1 The interaction of demands and co-worker support was also not significant in the FE models. Concerning the interplay of job demands, control and support underlying the JDCS model, there was no significant effect found in the models.

2 A three-way interaction of job demands, control and destructive leadership in the sense of the JDCS model was not significant neither in the OLS nor in the FE models (results not shown). In summary, control only played and an inferior role in the explanation of differences in employees’ work ability.
